# Performance of Polymerase Chain Reaction Techniques Detecting Perforin in the Diagnosis of Acute Renal Rejection: A Meta-Analysis

**DOI:** 10.1371/journal.pone.0039610

**Published:** 2012-06-29

**Authors:** Yushu Shang, Weiqiang Ju, Yuan Kong, Paul M. Schroder, Wenhua Liang, Xiaoting Ling, Zhiyong Guo, Xiaoshun He

**Affiliations:** 1 Organ Transplant Center, the First Affiliated Hospital, Sun Yat-sen University, Guangzhou, China; 2 Department of Medical Microbiology and Immunology, University of Toledo College of Medicine, Toledo, Ohio, United States of America; Northwestern University Feinberg School of Medicine, United States of America

## Abstract

**Background:**

Studies in the past have shown that perforin expression is up-regulated during acute renal rejection, which provided hopes for a non-invasive and reliable diagnostic method to identify acute rejection. However, a systematic assessment of the value of perforin as a diagnostic marker of acute renal rejection has not been performed. We conducted this meta-analysis to document the diagnostic performance of perforin mRNA detection and to identify potential variables that may affect the performance.

**Methodology/Principal Findings:**

Relevant materials that reported the diagnostic performance of perforin mRNA detection in acute renal rejection patients were extracted from electronic databases. After careful evaluation of the studies included in this analysis, the numbers of true positive, true negative, false positive and false negative cases of acute renal rejection identified by perforin mRNA detection were gathered from each data set. The publication year, sample origin, mRNA quantification method and housekeeping gene were also extracted as potential confounding variables. Fourteen studies with a total of 501 renal transplant subjects were included in this meta-analysis. The overall performance of perforin mRNA detection was: pooled sensitivity, 0.83 (95% confidence interval: 0.78 to 0.88); pooled specificity, 0.86 (95% confidence interval: 0.82 to 0.90); diagnostic odds ratio, 28.79 (95% confidence interval: 16.26 to 50.97); and area under the summary receiver operating characteristic curves value, 0.9107±0.0174. The univariate analysis of potential variables showed some changes in the diagnostic performance, but none of the differences reached statistical significance.

**Conclusions/Significance:**

Despite inter-study variability, the test performance of perforin mRNA detected by polymerase chain reaction was consistent under circumstances of methodological changes and demonstrated both sensitivity and specificity in detecting acute renal rejection. These results suggest a great diagnostic potential for perforin mRNA detection as a reliable marker of acute rejection in renal allograft recipients.

## Introduction

Renal transplantation has been the treatment of choice for patients with end-stage renal disease (ESRD) for decades. However, although novel and powerful immunosuppressive drugs have been developed, acute rejection (AR) remains a major cause of allograft dysfunction and allograft failure [1], [2]. Even a single episode of AR can be a strong predictor of graft failure [3].

Currently, the diagnosis of AR is established based on histological evaluation of allograft biopsy samples. However, biopsy is an invasive procedure that may cause biopsy-associated complications such as perirenal hematoma, hematuria and infection [4], [5], which restrict its application for serial surveillance testing. In addition, sampling error and the variability of the pathological changes of AR make it difficult to make definitive diagnoses based on renal biopsy in many cases [6]. Other methods such as ultrasonography and serum creatinine measurements can be indicative of ARbut cannot reach a conclusive diagnosis [7], [8]. Therefore, developing a reliable, specific and non-invasive diagnostic method for identifying ARwould be of great help to improve clinical practice in renal transplantation.

Since allograft infiltration by T lymphocytes is a distinctive feature of rejection, analyzing the expression of specific genes involved in T cell activation provides a new option for AR diagnosis. Among the numerous cell subsets that infiltrate the graft site, cytotoxic T lymphocytes (CTL) are one of the major effector cells during the AR response. Lipman et al. [9] and Suthanthiran et al. [10] revealed a significant increase in transcription of the gene encoding perforin, one of the predominant effector molecules of CTLs [11], in allograft biopsy samples from AR patients using polymerase chain reaction (PCR) techniques. Since that time, many studies had been conducted to validate this approach for AR diagnosis in the clinic, and the samples collected for analysis have been expanded from allograft biopsy samples to less invasive peripheral blood leukocytes (PBL) and urine samples.

Although increased levels of perforin mRNA were a common finding during AR in a series of studies [12]–[14], controversy still exists regarding the clinical utility of this test due to the single study design of the previous work and the variable laboratory methodology used to perform the test among the different studies. Herein, we performed a meta-analysis to document the diagnostic performance of perforin mRNA detection in the identification of AR and try to determine its clinical utility by seeking the potential variables that may affect the performance of this test. These data provide important insights that inform clinical physicians regarding the diagnosis of AR in renal transplantation.

## Materials and Methods

### Study Protocol

This analysis was conducted in accordance with a predetermined protocol following the recommendations of Deeks et al. [15]. The data collection and reporting were in accordance with Preferred Reporting Items for Systematic Reviews and Meta-Analyses: The PRISMA Statement (Table S1).

### Search Strategy

Relevant materials in the scientific literature were searched in electronic databases including MEDLINE, EMBASE and the Cochrane Database of Systematic Reviews prior to December 1st, 2011, without date or language limitations. The following combinations of key words were used to search for related studies: “perforin” AND (“renal transplant” OR “renal transplantation” OR “kidney transplant” OR “kidney transplantation”) AND “rejection.” The electronic searching was supplemented by checking reference lists from the identified articles for additional original reports.

### Inclusion and Exclusion Criteria

Studies were included if they fulfilled the following criteria: (1) Two comparison groups of patients were necessary for every study: AR group and non-rejection group. (2) Patient samples were diagnosed as AR or non-rejection based on the histological evidence according to Banff classification. (3) Quantitative detection of perforin mRNA expression level was accomplished by PCR techniques. (4) The mRNA detection was conducted either at the same time as biopsy pathological evaluation or immediately after with the samples being frozen for preservation during the evaluation. (5) The expression level of perforin was compared to the chosen housekeeping genes which were expressed at a constant level in samples from different patient groups. (6) A specific cutoff value was set to interpret the perforin mRNA results as positive or negative for AR (for those studies which defined the results as “detectable” or “undetectable,” “detectable” results were regarded as positive and vice versa).

The following types of studies were excluded from this meta-analysis: (1) Works designated as conference abstracts, letters, case reports, editorials or reviews. (2) Studies only involving pediatric patients.

### Assessment of Study Quality

The quality of each study’s methodology was assessed using the 14-item Quality Assessment of Diagnostic Accuracy Studies (QUADAS) list [16]. Each question was assigned with a response of yes, no, or unclear when evaluating each of the included studies. Since the assessment of quality related strongly to the reporting of results, a well conducted study could score poorly if the methods and results were not reported in sufficient detail. Therefore, we did not report the assessment in scores but in descriptive forms only.

Publication bias was tested using funnel plots and the Egger test by Stata statistical software (STATA) version 11.0 [17]. An asymmetric distribution of data points in the funnel plot and a quantified result of *P*<0.05 in the Egger test indicated the presence of potential publication bias [18].

### Data Extraction

The following data were extracted from each eligible study: year of publication, sample origin, mRNA quantification method, housekeeping gene, and the number of true positive (TP), false positive (FP), false negative (FN) and true negative (TN) cases of AR identified by perforin mRNA levels. All subjects who displayed biopsy results with any degree of AR defined by Banff classification were assigned to the rejection group, regardless of cellular or humoral rejection. The subjects with biopsies showing no evidence of any types of rejection, including normal tissues and tissues with non-rejection pathological changes, were assigned to the non-rejection group. The selected articles were assessed by two reviewers (YS and XL), independently. Disagreements were resolved by consultation with a third reviewer (ZG).

### Statistical Analysis

Data were analyzed using Meta-Disc Version 1.4 [19] and STATA version 11.0. The test performance of perforin mRNA detection for the identification of ARwas measured by the following indicators: sensitivity, specificity and diagnostic odds ratio (DOR). Sensitivity was represented by the proportion of AR cases that were correctly identified by the positive results of perforin mRNA levels. Specificity was represented by the proportion of non-rejection cases that were correctly identified by the negative results of perforin mRNA levels [20]. As different cutoff values were used in each study, there was the potential for a threshold effect which would affect the conclusions of this analysis. Therefore, it was more reliable to define the summary of test performance using DOR than simply pooling sensitivity and specificity together across the studies. DOR was an independent indicator ranging from 0 to infinity, which represented how much greater the odds of having AR were for patient with a positive perforin mRNA result than for patient with a negative perforin mRNA result. The higher the DOR, the better the discriminatory ability of the test was [21].

The summary receiver operating characteristic (SROC) curve was plotted based on the combination of sensitivity and specificity, and the area under the curve (AUC) value was then calculated as a global measurement of test performance. The closer the AUC was to 1, the better the test performance [22].

Because of potential heterogeneity between studies, effect sizes were pooled by random-effects models of DerSimonian and Laird in Meta Disc [23]. Empty cells were handled using a 0.5 continuity correction.

### Heterogeneity

The χ^2^ test was used to examine heterogeneity in pooling sensitivity and specificity. The Cochran Q test was used to examine heterogeneity in pooling DOR. Heterogeneity was considered to be statistically significant when *P*<0.05 in these qualitative tests. We also conducted the *I* ^2^ test in every pooling analysis to quantitatively estimate the proportion of total variation across studies that was attributable to heterogeneity rather than chance. The *I* ^2^ value would range from 0 to 100%, with a value over 50% indicating significant heterogeneity.

The existence of a threshold effect would manifest as a curvilinear shape in the SROC curves. In addition, we used a Spearman correlation analysis to confirm the absence or presence of a threshold effect by looking for an inverse relationship between sensitivity and specificity. A value of *P*<0.05 would indicate a significant threshold effect was present [24].

### Sensitivity Analysis

To determine whether any single study was incurring undue weight in the analysis, we systematically removed one set of study data and checked the pooled results for the remaining studies to see if they changed significantly. The sensitivity analysis was conducted for every study.

### Univariate Analysis

To identify the sources of potential heterogeneity that influenced the results of this analysis, a univariate analysis was conducted. Based on the literature review, the following factors were chosen as potential variables that may have influenced the test performance: year of publication, sample type, mRNA quantification method and housekeeping gene selection. Data sets were stratified based on these factors and the test performance would be compared between subgroups using the DOR values and the AUC of the SROC curves as the major parameters. The comparison was conducted using random-effects models in STATA. A value of *P*<0.05 in the comparison of DOR indicated a significant change in the test performance due to the covariate.

## Results

### Literature Search and Characteristics of the Included Studies

**Figure 1:**
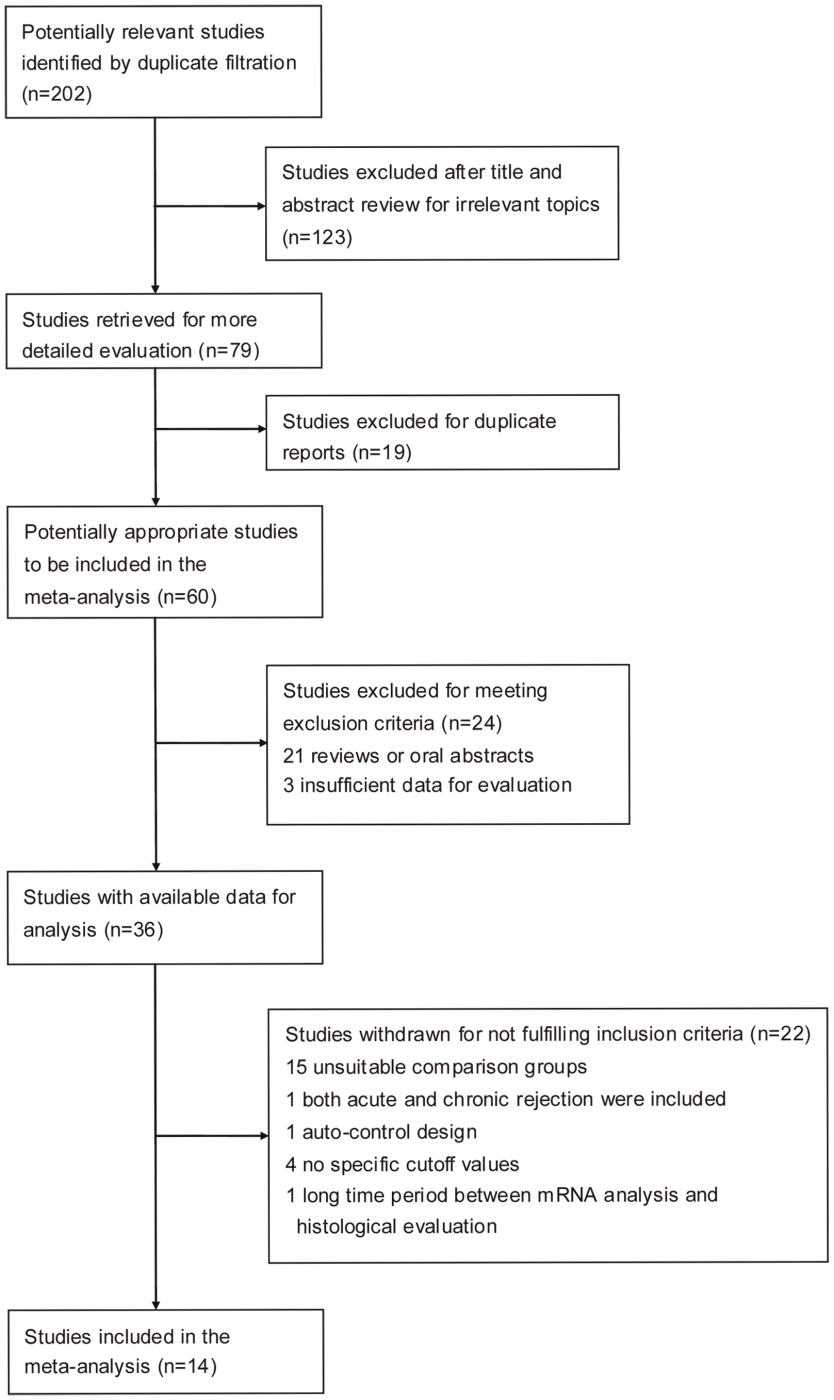
Flow chart describing the literature search conducted for this meta-analysis.

After the primary search of the electronic databases for published work on the subject, 202 studies were identified. Of these studies, 123 were excluded after further review of the title and abstract for irrelevant topics, and an additional 19 were excluded for duplication of the reports, which left 60 studies undergoing full text review. The detailed process of this literature search is shown in Figure 1.

After careful review, 14 studies with a total of 501 subjects were included in this meta-analysis. In 2 studies [25], [26], perforin expression was detected in both graft biopsy and PBL samples. In another study [27], perforin expression was detected in both PBL and urine samples. We decided to retrieve each group as an independent data set for a total of 17 data sets included in this analysis. The characteristics of each included study are shown in Table 1.

**Table 1:** Study characteristics of each included study.

Reference number	Author	Publication year	Sample origin	Messenger RNA quantification method	Housekeeping gene	Number of subjects	Test results			
							TP	FP	FN	TN
9	Lipman et al.	1994	graft biopsy	competitive RT-PCR	GAPDH	26	9	2	1	14
25	Netto et al.	2002	graft biopsy	RT-PCR	β-actin	29	4	1	3	21
			PBL	RT-PCR	β-actin	29	6	0	1	22
26	Vasconcellos et al.	1998	graft biopsy	competitive RT-PCR	GAPDH	31	11	2	0	18
			PBL	competitive RT-PCR	GAPDH	31	9	3	2	17
27	Dias et al.	2008	PBL	RT-PCR	cyclophilin	48	20	7	0	21
			urine	RT-PCR	cyclophilin	50	20	4	0	26
28	Sabek et al.	2002	PBL	RT-PCR	18s rRNA	27	5	5	3	14
29	Lipman et al.	1998	graft biopsy	competitive RT-PCR	GAPDH	21	6	0	5	10
30	Strehlau et al.	1997	graft biopsy	competitive RT-PCR	GAPDH	27	12	1	3	11
31	Li et al.	2001	urine	RT-PCR	cyclophilin	44	20	3	4	17
32	Øzbay et al.	2009	urine	real-time quantitative RT-PCR	cyclophilin	41	21	4	3	13
33	Galante et al.	2006	urine	real-time quantitative RT-PCR	cyclophilin	24	11	1	2	10
34	Shin et al.	2005	PBL	competitive RT-PCR	β-actin	15	5	1	2	7
35	Simon et al.	2003	PBL	real-time quantitative RT-PCR	18s rRNA	16	4	0	1	11
36	Dugr’e et al.	2000	PBL	RT-PCR	β-actin	21	4	1	4	12
37	Dias et al.	2004	graft biopsy	RT-PCR	GAPDH	21	10	5	1	5

Abbreviations: TP, true positive; FP, false positive; FN, false negative; TN, true negative; PBL, peripheral blood leukocyte; RT-PCR, reverse transcription polymerase chain reaction; GAPDH, glyceraldehyde-3-phosphate dehydrogenase.

### Study Quality

We used the QUADAS list of questions to review the test quality of the included studies. Most of the studies satisfied a majority of the items on the QUADAS list. The most common missing items in the studies included in this analysis were reports of intermediate results and withdrawn cases. In addition, some of the studies failed to mention the blinded interpretations between the PCR results and the histological evaluation (Table S2).

The Egger test revealed the possibility of significant publication bias among the included reports (*P = *0.008). The funnel plot in Figure S1 also presented a certain degree of asymmetry, indicating the potential for publication bias among the studies included in this analysis.

### Overall Diagnostic Performance of Perforin Expression

**Figure 2:**
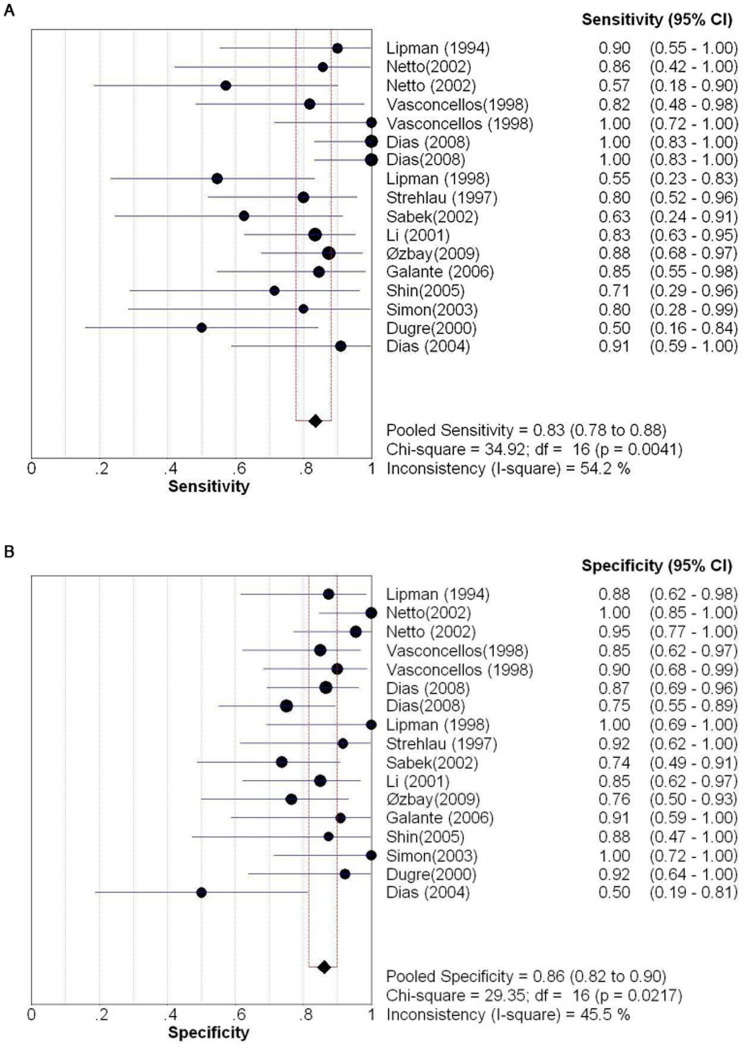
Sensitivity and specificity of perforin mRNA detection for the diagnosis of AR. (A) Pooled sensitivity. (B) Pooled specificity. Effect sizes were pooled by random-effects models. The point estimates from each study are shown as solid squares. The pooled estimates are shown as a solid diamond. Error bars represent 95% CIs.

Figure 2 shows the overall diagnostic measurements of perforin expression in detecting AR. The summary sensitivity was 0.83 [95% confidence interval (CI): 0.78 to 0.88], with individual sensitivities ranging from 0.50 to 1.00. The summary specificity was 0.86 (95% CI: 0.82 to 0.90), with individual specificities ranging from 0.50 to 1.00. Both pooled estimations showed significant heterogeneity (Sensitivity: *P* = 0.0041, *χ* ^2^ = 34.92, *I* ^2^ = 54.2%; specificity: *P = *0.022, *χ* ^2^ = 29.35, *I* ^2^ = 45.5%).

**Figure 3:**
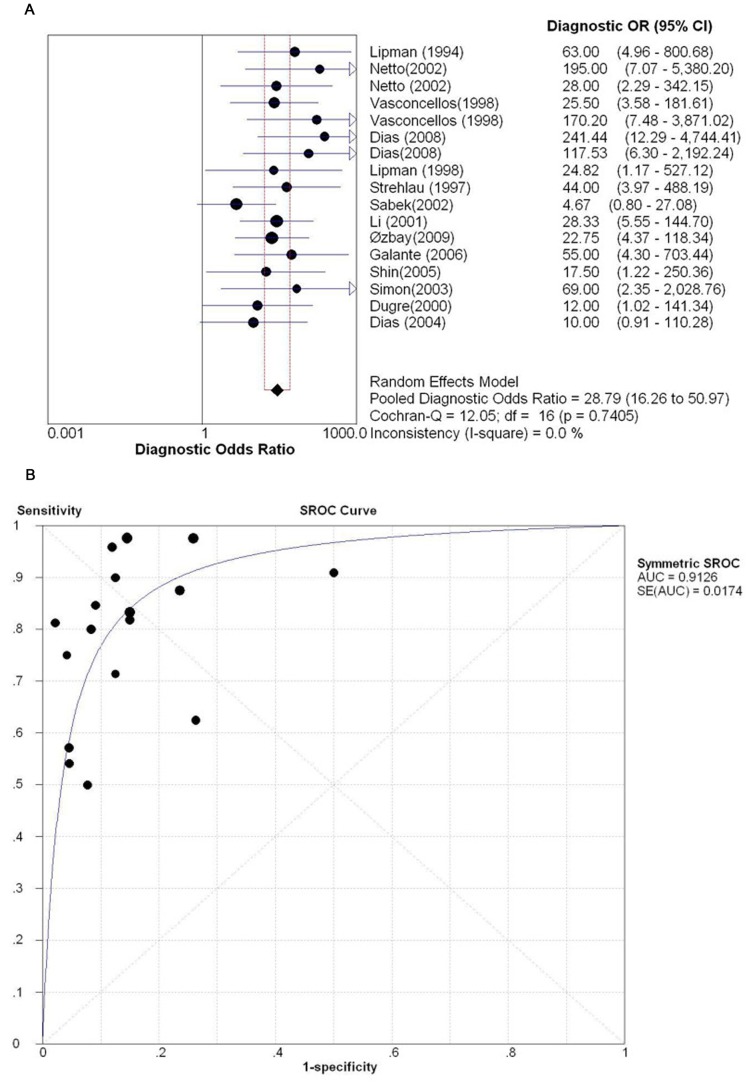
Overall DOR and SROC curves for all data sets describing the diagnostic performance of perforin mRNA detection in identifying AR. (A) Overall DOR. (B) The SROC curves for all data sets. Effect sizes were pooled by random-effects models. The pooled DOR is shown as a solid diamond. Each square in the SROC curve represents one study. Sample size is indicated by the size of the square.

The pooled DOR and the SROC curves based on summary sensitivity and specificity across all data sets are shown in Figure 3. The pooled DOR was 28.79 (95% CI: 16.26 to 50.97), with individual DORs ranging from 4.67 to 241.44. The results of DOR showed consistency accross the included reports, without noticeable heterogeneity (*P = *0.74, Cochran-Q = 12.05, *I* ^2^ = 0.0%). The point size in the SROC curve represented the proportional study weight. Most data gathered near the top left corner where sensitivity and specificity were both the highest. The AUC value was 0.9107±0.0174.

Although we did not notice a curvilinear shape distribution of the data in the SROC curve, the Spearman correlation analysis revealed a significant result (*P = *0.032), suggesting the potential presence of a threshold effect.

### Sensitivity Analysis

We systematically removed one data set at a time and recalculated the DOR and AUC values for the remaining studies. The largest change occurred when removing the data set from Sabek et al. [28], which changed the pooled DOR from 28.79 to 35.68 (+23.9%), and the corresponding change in AUC value was from 0.9107 to 0.9228 (+1.33%). The second largest change occurred when removing the urine subgroup from Dias et al. [27], which changed the pooled DOR from 28.79 to 26.54 (−7.81%) and the corresponding AUC value from 0.9107 to 0.9058 (−0.54%). These results indicated that no single data set carried enough weight to significantly influence the pooled test performance reported for the ability of perforin mRNA detection to identify cases of acute renal rejection.

### Univariate Analysis

#### Publication year

Based on the year of publication of the studies included in this analysis, we divided the data sets into two subgroups: those reported prior to the year 2000 and those reported after the year 2000 (including studies published in 2000). This time point was chosen because significant progress was made in the PCR technology and experimental methodology at the beginning of the 21st century, which may have had an effect on the perforin mRNA detection performance. We noticed a remarkable difference in the amount of publications in each subgroup. Only 4 reports were published prior to 2000 [9], [26], [29], [30], one of them contained 2 data sets which made it 5 data sets in this subgroup. The remaining 10 reports (12 data sets) were published after 2000 [25], [27], [28], [31]–[37]. The DOR of studies before the year 2000 was 43.52 while the DOR of studies after 2000 was 24.90. The difference was not statistically significant (*P = *0.59).

#### Sample origin

The studies were stratified according to the 3 types of samples: allograft biopsy tissue, PBL and urine. The biopsy subgroup contained 6 data sets [9], [25], [26], [29], [30], [37], the PBL subgroup contained 7 data sets [25]–[27], [28], [34]–[36], and the urine subgroup contained 4 data sets [27], [31]–[33]. The DORs were 35.11, 21.32, 36.76 for biopsy group, PBL group and urine group, respectively. However, the difference in DORs did not reach a level of statistical significance (*P = *0.77).

#### Messenger RNA quantification method

There were 3 different PCR techniques used in the included studies to quantify perforin mRNA: 8 data sets used reverse transcriptase PCR (RT-PCR) [25], [27], [28], [31], [36], [37], 6 data sets used competitive RT-PCR [9], [26], [29], [30], [34], and 3 data sets used real-time quantitative RT-PCR [32], [33], [35]. The DOR was 25.23 for RT-PCR, 37.94 for competitive RT-PCR and 33.35 for real-time quantitative RT-PCR. The difference between the three techniques was not statistically significant (*P = *0.89).

#### Housekeeping gene

Four different housekeeping genes were used as the standard expression in the included studies to measure the relative expression level of perforin: cyclophilin for 4 data sets [27], [31]–[33], β-actin for 5 [25], [27], [34], [36], 18s rRNA for 2 [28], [35], and glyceraldehyde-3-phosphate dehydrogenase (GAPDH) for 6 [9], [26], [29], [30], [37]. The DOR was 36.76 for cyclophilin group, 34.38 for β-actin group, 12.03 for 18s rRNA group and 33.45 for GAPDH group. However, the result was not statistically significant either (*P = *0.95).

Table 2 summarizes the results of univariate analysis.

**Table 2:** Univariate analysis of potential variables influencing the test performance of perforin during AR.

Variables	Subgroups	Number of independent data sets		Sensitivity (95% CI)	Specificity (95% CI)	DOR (95% CI)	AUC
**Publication year**	before 2000	5		0.81 (0.69–0.90)	0.90 (0.81–0.95)	43.52 (14.19–133.46)	0.9406
	after 2000	12		0.84 (0.78–0.90)	0.85 (0.79–0.89)	24.90 (12.82–48.37)	0.9028
**Sample origin**	graft biopsy	6		0.80 (0.68–0.89)	0.88 (0.79–0.94)	35.11 (12.02–102.56)	0.9210
	PBL	7		0.80 (0.69–0.89)	0.86 (0.78–0.92)	21.32 (7.97–57.09)	0.9028
	urine	4		0.89 (0.80–0.95)	0.85 (0.75–0.92)	36.76 (13.59–99.39)	0.9158
**PCR techniques**	RT-PCR	8		0.85 (0.76–0.91)	0.84 (0.78–0.89)	25.23 (9.59–66.37)	0.9056
	competitive RT-PCR	6		0.80 (0.68–0.89)	0.90 (0.81–0.95)	37.94 (13.51–106.56)	0.9370
	real-time quantitative RT-PCR	3		0.86 (0.71–0.95)	0.87 (0.73–0.96)	33.35 (9.26–120.09)	0.9196
**Housekeeping gene**	cyclophilin	4		0.89 (0.80–0.95)	0.85 (0.75–0.92)	36.76 (13.59–99.39)	0.9158
	β-actin	5		0.80 (0.66–0.90)	0.89 (0.81–0.95)	34.38 (10.16–116.35)	0.9372
	18s rRNA	2		0.69 (0.39–0.91)	0.83 (0.65–0.94)	12.03 (0.96–151.24)	unavailable a
	GAPDH	6		0.83 (0.72–0.91)	0.85 (0.76–0.92)	33.45 (12.12–92.34)	0.9194
**All**			17	0.83 (0.78–0.88)	0.86 (0.82–0.90)	28.79 (16.26–50.97)	0.9107

a Three independent data points are required at least to draw an SROC curve.

Abbreviations: DOR, diagnostic odds ratio; CI, confidence interval; AUC, area under the curve of the SROC curve; PBL, peripheral blood leukocyte; PCR, polymerase chain reaction; RT-PCR, reverse transcription polymerase chain reaction; GAPDH, glyceraldehyde-3-phosphate dehydrogenase.

## Discussion

Since the middle of the 20^th^ century, great success has been made in renal transplantation with the progress in surgical techniques, expanded organ sources, organ preservation techniques, novel immunosuppressants and management of complications. However, transplant patients are still facing many challenges, among which AR draws the greatest attention. Despite the fact that histological evaluation for AR has been well defined in guidelines such as Banff criteria [38] and Cooperative Clinical Trials in Transplantation (CCTT) criteria [39], novel and less invasive methods are still required to improve the diagnostic evaluation of AR. The effector molecules of CTLs such as perforin, granzyme B, Fas and Fas ligand are potential diagnostic markers for AR, especially when they can be detected in samples such as PBLs and urine that do not require invasive procedures to obtain. The major objectives of conducting this meta-analysis were to explore the diagnostic performance of perforin mRNA expression in AR and to determine its clinical utility. To our knowledge, this is the first pooled estimation of the diagnostic performance of perforin mRNA detection for the evaluation of AR in renal transplant recipients.

In this meta-analysis, we included 14 relevant studies with a total of 501 subjects. Although results were not consistent across the different studies, the overall diagnostic performance of detecting perforin mRNA in kidney transplant patients showed pooled sensitivity and specificity of 0.83 (95% CI: 0.78 to 0.88) and 0.86 (95% CI: 0.82 to 0.90), respectively. The pooled DOR and AUC of the SROC curves for all data sets were 28.79 (95% CI: 16.26 to 50.97) and 0.9107±0.0174, respectively. These results represented a good diagnostic efficacy for perforin mRNA detection in identifying AR, regardless of the sample origin and methodology variation. Furthermore, to investigate potential variables that might have influenced the diagnostic performance, we conducted a univariate analysis trying to provide clues for methodology standardization. In this analysis, none of the chosen factors appeared to have a significant effect on the diagnostic performance. This lack of variation from the chosen factors may be due to the small sample sizes of the included data sets since this diagnostic method had not been widely used in transplant centers. In addition, the perforin gene sequences used in the included reports were not uniform, which could be another potentail source of variation that may have influenced test performance. However, the limited number of data sets using each perforin sequence restricted us from categorizing the studies into subgroups for the univariate analysis.

In several clinical studies during the 1990s [9], [10], [30], cytotoxic gene expression was found to be up-regulated in allografts during AR. However, these discoveries had limited impact as diagnostic tests that supplemented the histological diagnosis of AR at that time. More recently, given the fact that lymphocytes would infiltrate the kidneys during AR and present in urine sediment cells, Li et al. [31] explored the utilization of perforin mRNA detection in urine cells as a non-invasive diagnostic marker of AR. Subsequent studies conducted in other centers confirmed the feasibility of this approach [32], [33]. Although in our analysis, the diagnostic performance of urine sample didn’t stand out particularly, the result was still encouraging since it brought hope for a non-invasive method for the diagnosis of AR which was as reliable as biopsy sample.

Debates about the application of urine perforin detection mainly focus on the differential diagnosis between rejection and other complications such as delayed graft function (DGF) and urinary tract infection (UTI). In the study conducted by Yannaraki et al. [14], an increase in perforin mRNA was found in both the AR group and the UTI group. Their experience suggested a significant overlap of perforin mRNA levels in different clinical conditions, which made it difficult to establish a threshold value for differential diagnosis. Øzbay et al. [32] reported similar results when trying to differentiate AR from bacteriuria. This may be explained by the similar cytolytic response of the activated T lymphocytes during both rejection and infection. In three of the 4 included studies in the urine subgroup of this meta-analysis [31]–[33], the non-rejection samples were composed of stable grafts only, while in the other study [27], the non-rejection samples contained chronic allograft nephropathy, toxic tubulopathy, nonspecific changes, acute tubular necrosis and renal-vein thrombosis. The non-rejection controls in other included studies also contained samples with multiple other types of kidney dysfunction other than graft rejection, which did not allow us to carry out a meta-analysis of the differential diagnostic performance of perforin. Therefore, we could not conclude that a high perforin expression level would definitely point to the diagnosis of AR, which would require supplemental laboratory tests to rule out other complications.

Although urine is the ideal choice for a non-invasive procedure, there are some potential limitations to this approach. Most importantly, the test depends on urine production. This limits the utilization in patients under anuric conditions which can appear during AR, acute tubular necrosis (ATN), DGF, as well as other conditions. In the study conducted by Dias et al. [27], nearly 20% of the patients were unable to provide sufficient urine samples for analysis. Given these circumstances, the evaluation of perforin mRNA levels in PBLs and allografts are important alternatives. An increase in perforin mRNA can help clinical decision-making for early enhanced immunosuppression intervention before histological evidence of substantial damage develops, and a decrease in perforin mRNA levels may provide an indication of response to therapy.

There are several limitations in this meta-analysis. First, qualities of the included studies were not uniform. The essential demographical data like age and gender distributions were missing in some studies, which might be a potential heterogeneity source in the analysis. Also, the specific cut-off values for the mRNA level were not provided in most of the studies. In addition, only 14 studies met the inclusion criteria in this analysis. The small sample size limited the generalization of the results and did not allow us to test the differential diagnostic performance of perforin mRNA detection. All these limitations provide room for future evaluation.

In conclusion, the test performance of perforin mRNA detected by PCR techniques was impressive and consistent under circumstances of methodological changes. The test in urine stood out as a potential novel and non-invasive method for the reliable diagnosis of AR, or at least as an indicator that a biopsy is warranted. Prospective studies with larger sample sizes would reinforce the findings revealed in the current meta-analysis and may be able to reveal how perforin mRNA detection would help to differentiate between diagnoses that are clinically similar to AR, providing more conclusive evidence for its clinical utility in the evaluation of renal transplant recipients.

## Supporting Information

Figure S1 **Funnel plot for the assessment of potential publication bias.** The funnel graphs plot the log of the DOR against the standard error (SE) of the log of the DOR. Each solid circle represents each study in the meta-analysis. Asymmetry of the circle distribution between two sides indicates potential publication bias.
(TIF)

Table S1 **PRISMA 2009 check list.**
(DOC)

Table S2 **Quality assessment of the included articles.** Abbreviation: QUADAS, Quality Assessment of Diagnostic Accuracy Studies.
(DOC)
