# Supplementary figures and images for: Performance of Polymerase Chain Reaction Techniques Detecting Perforin in the Diagnosis of Acute Renal Rejection: A Meta-Analysis
Source: PLoS One. 2012 Jun 29;7(6):e39610. doi: 10.1371/journal.pone.0039610 (PMC3387236; doi:10.1371/journal.pone.0039610)

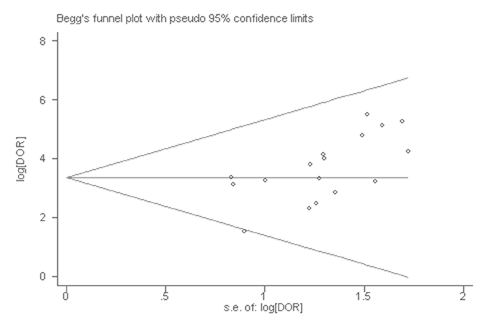

Supplement: Figure S1 — Funnel plot for the assessment of potential publication bias. The funnel graphs plot the log of the DOR against the standard error (SE) of the log of the DOR. Each solid circle represents each study in the meta-analysis. Asymmetry of the circle distribution between two sides indicates potential publication bias. (TIF) [file pone.0039610.s001.tif]
