# Supplementary material for: Performance of Polymerase Chain Reaction Techniques Detecting Perforin in the Diagnosis of Acute Renal Rejection: A Meta-Analysis
Source: PLoS One. 2012 Jun 29;7(6):e39610. doi: 10.1371/journal.pone.0039610 (PMC3387236; doi:10.1371/journal.pone.0039610)
Supplement: Table S2 — Quality assessment of the included articles. Abbreviation: QUADAS, Quality Assessment of Diagnostic Accuracy Studies. (DOC) [file pone.0039610.s003.doc]

**Table S2**. Quality assessment of the included articles.

| **QUADAS list item** | **Reference number of the included studies** | | | | | | | | | | | | | | |
| --- | --- | --- | --- | --- | --- | --- | --- | --- | --- | --- | --- | --- | --- | --- | --- |
|  | 9 | 25 | 26 | 27 | 28 | 29 | 30 | 31 | 32 | 33 | 34 | 35 | 36 | 37 |  |
| 1. Did the spectrum of patients represent the patients who will receive the test in practice? | + | + | + | + | + | + | + | + | + | + | + | + | + | + |  |
| 2. Were selection criteria clearly described? | + | + | + | + | + | + | + | + | + | + | + | + | + | + |  |
| 3. Is the reference standard likely to correctly classify the target condition? | + | + | + | + | + | + | + | + | + | + | + | + | + | + |  |
| 4. Is the period between the reference standard and index test short enough to be reasonably sure that the target condition did not change between the 2 tests? | + | + | + | + | + | + | + | + | + | + | + | + | + | + |  |
| 5. Did the entire sample or a random selection of the sample receive verification using a reference standard of diagnosis? | + | + | + | + | + | + | + | + | + | + | + | + | + | + |  |
| 6. Did patients receive the same reference standard regardless of index test result? | + | + | + | + | + | + | + | + | + | + | + | + | + | + |  |
| 7. Was the reference standard independent of the index test (i.e., index test did not form part of the reference standard)? | + | + | + | + | + | + | + | + | + | + | + | + | + | + |  |
| 8. Was execution of the index test described in sufficient detail to permit replication of the test? | + | + | + | + | + | + | + | + | + | + | + | + | + | + |  |
| 9. Was execution of the reference standard described in sufficient detail to permit its replication? | + | + | + | + | + | + | + | + | + | + | + | + | + | + |  |
| 10. Were index test results interpreted without knowledge of results of the reference standard? | 0 | 0 | + | 0 | 0 | 0 | 0 | 0 | 0 | 0 | 0 | 0 | 0 | 0 |  |
| 11. Were reference standard results interpreted without knowledge of results of the index test? | + | 0 | 0 | 0 | + | 0 | 0 | + | 0 | 0 | 0 | 0 | 0 | + |  |
| 12. Were the same clinical data available when test results were interpreted as would be available when the test is used in practice? | + | + | + | + | + | + | + | + | + | + | + | + | + | + |  |
| 13. Were uninterruptable/intermediate test results reported? | - | - | - | - | - | - | - | - | - | - | - | - | - | - |  |
| 14. Were withdrawals from the study explained? | - | - | - | - | - | - | - | - | - | - | - | + | + | + |  |

Abbreviation: QUADAS, Quality Assessment of Diagnostic Accuracy Studies.
